# Associations of vomiting and antiemetic use in pregnancy with levels of circulating GDF15 early in the second trimester: A nested case-control study

**DOI:** 10.12688/wellcomeopenres.14818.1

**Published:** 2018-09-21

**Authors:** Clive J. Petry, Ken K. Ong, Keith A. Burling, Peter Barker, Sandra F. Goodburn, John R.B. Perry, Carlo L. Acerini, Ieuan A. Hughes, Rebecca C. Painter, Gijs B. Afink, David B. Dunger, Stephen O'Rahilly

**Affiliations:** 1 Department of Paediatrics, University of Cambridge Addenbrooke's Hospital Cambridge, Cambridge, CB2 0QQ, UK; 2 Medical Research Council Epidemiology Unit, University of Cambridge Addenbrooke's Hospital Cambridge, Cambridge, CB2 0QQ, UK; 3 NIHR Biomedical Research Centre Core Biochemistry Assay Lab, University of Cambridge Addenbrooke's Hospital Cambridge, Cambridge, CB2 0QQ, UK; 4 Department of Clinical Biochemistry, Addenbrooke's Hospital, Cambridge, CB2 0QQ, UK; 5 Department of Gynaecology and Obstetrics, Academic Medical Center of the University of Amsterdam, Amsterdam, 1105 AZ, The Netherlands; 6 Reproductive Biology Laboratory, Academic Medical Center of the University of Amsterdam, Amsterdam, 1105 AZ, The Netherlands; 7 Metabolic Research Laboratories and MRC Metabolic Diseases Unit, University of Cambridge Addenbrooke's Hospital Cambridge, Cambridge, CB2 0QQ, UK

**Keywords:** antiemetics, nausea, obesity, pregnancy, maternal-fetal relations

## Abstract

**Background:** Although nausea and vomiting are very common in pregnancy, their pathogenesis is poorly understood. We tested the hypothesis that circulating growth and differentiation factor 15 (GDF15) concentrations in early pregnancy, whose gene is implicated in hyperemesis gravidarum, are associated with nausea and vomiting.

**Methods: **Blood samples for the measurement of GDF15 and human chorionic gonadotrophin (hCG) concentrations were obtained early in the second trimester (median 15.1 (interquartile range 14.4-15.7) weeks) of pregnancy from 791 women from the Cambridge Baby Growth Study, a prospective pregnancy and birth cohort. During each trimester participants completed a questionnaire which included questions about nausea, vomiting and antiemetic use. Associations with pre-pregnancy body mass indexes (BMI) were validated in 231 pregnant NIPTeR Study participants.

**Results:** Circulating GDF15 concentrations were higher in women reporting vomiting in the second trimester than in women reporting no pregnancy nausea or vomiting: 11,581 (10,977-12,219) (n=175) vs. 10,593 (10,066-11,147) (n=193) pg/mL, p=0.02). In women who took antiemetic drugs during pregnancy (n=11) the GDF15 levels were also raised 13,157 (10,558-16,394) pg/mL (p =0.04). Serum GFD15 concentrations were strongly positively correlated with hCG levels but were inversely correlated with maternal BMIs, a finding replicated in the NIPTeR Study.

**Conclusions: **Week 15 serum GDF15 concentrations are positively associated with second trimester vomiting and maternal antiemetic use in pregnancy. Given GDF15’s site of action in the chemoreceptor trigger zone of the brainstem and its genetic associations with hyperemesis gravidarum, these data support the concept that GDF15 may be playing a pathogenic role in pregnancy-associated vomiting.

## Introduction

Nausea and vomiting in pregnancy (NVP) affects 70–90% of all pregnant women. The most severe form of NVP, hyperemesis gravidarum (HG), leads to maternal dehydration and electrolyte imbalance and is the most common cause of hospital admission during early pregnancy ^1^. Even though the majority of cases of NVP are mild or moderate with little impact upon maternal well-being, HG has substantial consequences for the mother’s quality of life ^2^, psychological morbidity ^3^, workplace productivity ^4^ and decreased caloric intake for the mother ^5, 6^. Furthermore, HG may have potential adverse effects on the developing fetus, as indicated by higher likelihood of low birth weight, preterm delivery, and small size at birth for gestational age in women with HG ^7^. While effective pharmacological interventions are available, there are concerns regarding possible fetal teratogenicity of some agents ^8^.

The pathogenesis of HG is poorly understood. Primiparity, younger maternal age, non-smoking ^1^ and being underweight ^9– 11^ may be risk factors. Reproductive hormones, such as human chorionic gonadotropin (hCG), progesterone and estrogen, have been implicated due to their rise in concentrations in the mother’s circulation contemporaneous with the manifestation of NVP ^12^. However nausea and vomiting are not common side-effects of such agents when administered in other settings, nor are increases in reproductive hormones consistently associated with increased HG severity or duration ^13^. A family history of HG leads to a 3-fold increase in HG among the female offspring ^14^, which has led to the hypothesis that it may be genetically driven. Recent studies of HG have tentatively implicated rare variants in *TSHR*, which encodes the thyrotropin receptor ^15^, and *RYR2* ^16^, which encodes a stress-induced intracellular calcium release channel in some familial cases. Evolutionary theories have been proposed for NVP as a beneficial strategy to protect the fetus from maternal ingestion of noxious substances, particularly during the early stages of pregnancy, coinciding with organogenesis, when the fetus is most vulnerable ^12^.

Growth and Differentiation Factor 15 (GDF15) signaling through its receptor (a heterodimer of proteins coded for by the GDNF family receptor α–like ( *GFRAL*) and Rearranged During Transfection ( *RET*) genes) has recently been identified to activate the mammalian chemoreceptor trigger zone of the medulla to suppress food intake in mice ^17– 20^ and primates ^21^. As such it therefore represents a potential mechanism for the aversion to foods and eating behaviors during periods of stress, sickness or high vulnerability to external toxins ^12^. In the non-pregnant state *GDF15* is expressed at low levels in many tissues. In pregnancy, *GDF15* is highly expressed in the placenta from early time points. In standard pregnancies circulating levels rise rapidly in maternal blood during the first trimester of pregnancy and remain elevated until delivery ^22^. A genome wide association study has recently shown that variants in and around the *GDF15* locus are strongly associated with the risk of HG in pregnancy ^23^.

To explore the hypothesis that NVP might relate to circulating GDF15 levels, we measured serum GDF15 in Cambridge Baby Growth Study samples obtained from women who had been prospectively followed throughout their pregnancies. They had answered questionnaires in each of the three trimesters which had incorporated questions regarding nausea, vomiting and antiemetic use. As previous research has variably implicated hCG in the pathogenesis of NVP ^24^ we examined the relationships between hCG levels, NVP symptoms and GDF15 concentrations in those women in whom these measures were available. As there have been reports that low pre-pregnancy body mass index (BMI) predisposes to NVP ^9^ we also examined the relationship between pre-pregnancy BMI, GDF15 levels and NVP.

## Methods

### Cohort 1: Cambridge Baby Growth Study

The prospective Cambridge Baby Growth Study recruited 2,229 mothers (and their partners and offspring) attending antenatal ultrasound clinics during early pregnancy at the Rosie Maternity Hospital, Cambridge, United Kingdom, between 2001-9 ^25^. All mothers were over 16 years of age. Pre-pregnancy weight and height were self-reported. In this cohort, 96.9% of the offspring were of white ethnicity, 0.8% were of mixed race, 0.6% were black (African or Caribbean), 0.8% were East-Asian, and 0.9% were Indo-Asian. Research blood samples, from which serum was separated and aliquoted, were collected from 1,177 (52.8%) mothers at recruitment (median 15.0 weeks, interquartile range 1.6 weeks). Around week 14 of pregnancy the participants were offered the chance to have routine blood taken for the measurement of serum alpha-fetoprotein (AFP), hCG and unconjugated estriol (uE3) as the pre-natal screening triple test.

Each mother was given a printed questionnaire at recruitment to fill in and return after the birth of their child ^26^. The participants were encouraged to fill their questionnaire in as their pregnancy progressed. It included boxes to tick if the participants had experienced NVP during pregnancy ^27^. If either the nausea or vomiting boxes was ticked there were further boxes to complete concerning the timing (i.e. week(s) of pregnancy) when the nausea or vomiting was experienced. An additional question asked “Have you taken any medicine during this pregnancy?” and a table was provided for positive responses with the following headings: “Name”, “Disease”, “Daily Dose”, “No. of Days” and “Gestational Week(s)”. A total of 1,238 women (54.6%) returned a questionnaire. Of these, only 3 self-reported that they had HG and a further 17 reported treatment with an antiemetic agent: cyclizine (n=7), promethazine (n=5), prochlorperazine (n=4), metoclopramide (n=2), domperidone (n=2), prednisolone (n=2), chlorphenamine (n=1), ondansetron (n=1), chlorpromazine (n=1) and unknown (n=1). The timing of NVP was categorized into trimesters (first: up to gestational week 12; second: 13 to 27 weeks; third: 28+ weeks).

The current analysis was based on 791 women in the Cambridge Baby Growth Study who had an available serum sample collected between gestational age 12 and 18 weeks and returned a completed questionnaire ^26, 27^. Of these there were only 11 women who reported taking antiemetics during pregnancy. These women were representative of the whole cohort by having similar maternal pre-pregnancy BMIs, parities and offspring birth weights (adjusted for standard confounders) to those women who did not return a pregnancy questionnaire ( Supplementary Table 1).

### Cohort 2: NIPTeR Study

The Non-Invasive Prenatal RNA profiling in pregnancy (NIPTeR) study was set up to research the early detection of preeclampsia before symptoms emerge. Included were pregnant women of at least 18 years old at their first antenatal visit. Enrolment took place between September 2015 and November 2017 at the Academic Medical Center, Amsterdam, The Netherlands. Antiemetic use, history of hospital admissions for HG, and pre-pregnancy weight and height were retrieved from the medical charts. In addition to the routine blood samples, a blood sample for the NIPTeR Study was taken. Within 6 hours of blood collection plasma was isolated by a 2-step protocol: first a low speed platelet-rich plasma separation, followed by a general high-speed step for clearance of all cells.

The current analysis was based on data from 231 women whose blood was sampled between 10 and 18 weeks of pregnancy who did not report developing HG/antiemetic use.

### Ethics

The Cambridge Baby Growth Study was approved by the Cambridge Research Ethics Committee, Cambridge, United Kingdom (LREC 00/325). All procedures followed were in accordance with Good Clinical Practice guidelines. Written informed consent was obtained from all the study participants. The NIPTeR study was approved by the Academic Medical Center ethics committee (reference 2015_072) and all participating women provided written informed consent.

### Assays

GDF15 concentrations were measured in serum (Cambridge Baby Growth Study) and EDTA plasma (NIPTeR) using an in-house Meso Scale Discovery electrochemiluminescence immunoassay (Meso Scale Diagnostics, Rockville, Maryland, U.S.A.) developed using antibodies from R & D Systems Quantikine reagents (BioTechne Ltd., Abingdon, U.K.). The sensitivity of this assay was 3 pg/mL and the working range went up to 32,000 pg/mL. Batch-to-batch variability was 9.8% at 352 pg/mL, 8.1% at 1490 pg/mL and 7.8% at 6667 pg/mL. Pre-natal screening assays were performed using routine AutoDELFIA time-resolved fluoroimmunoassays (PerkinElmer Life Sciences, Wallac Oy, Turku) and the results were expressed as multiples of the median (MOM) ^28^.

### Statistical analysis

Women in the Cambridge Baby Growth Study were categorized into one of three groups: vomiting (independent of whether they reported having experienced nausea or not); nausea but no vomiting; and no nausea or vomiting ^27^. The primary outcome was vomiting during the second trimester, as this coincided with the timing of maternal serum sampling. These were compared to concentrations in women who reported no nausea or vomiting. Maternal pre-pregnancy BMI was calculated dividing body weight prior to pregnancy by height-squared. NIPTeR Study samples were used to validate the relationship between GDF15 and BMI.

Serum GDF15 concentrations were natural logarithm-transformed to achieve a normal distribution and were considered as the dependent variable in linear regression models with adjustment for gestational age at serum sample collection. Where the relationship with adjusted log-transformed GDF15 concentrations did not appear to be linear, data were transformed to approximate linearity prior to analysis (e.g. the reciprocal BMI was used). Statistical analyses were performed using Stata 13.1 (StataCorp LP, College Station, Texas, U.S.A.). P<0.05 was considered to indicate statistical significance.

## Results

### Maternal nausea and vomiting in pregnancy

37.7% (n=298) of the Cambridge Baby Growth Study women reported vomiting during any trimester of pregnancy. A further 37.9% (n=300) reported nausea but no vomiting, and only 24.4% (n=193) of the Cambridge Baby Growth Study women reported no nausea or vomiting. More women (32.0%, n=253) reported vomiting during the first trimester compared to 22.1% (n=175) in the second trimester, with only 3.8% (n=30) in the third trimester. 86.9% and 56.7% of those reporting vomiting in the second and third trimesters also reported vomiting during the first trimester, respectively. Women who reported vomiting during the second trimester were younger and were carrying relatively more female babies than women who reported no nausea or vomiting during pregnancy ( Table 1), but there were no differences in pre-pregnancy BMI.

**Table 1.** Clinical characteristics of the women who reported vomiting during the second trimester of pregnancy. This table shows comparisons of clinical characteristics between those women that reported vomiting during the second trimester of pregnancy and those women that reported no nausea or vomiting throughout pregnancy in the Cambridge Baby Growth Study. Those women who reported second trimester vomiting were very slightly younger and were carrying a higher proportion of female babies. There were no apparent differences in BMI, parity or prevalence of twin pregnancies however.

	Vomiting (2 ^nd^ trimester)	No nausea or vomiting	p-value
n	175	193	
Age at delivery (years)	32.8 (32.1-33.5)	33.7 (33.1-34.3)	0.047
BMI (kg/m ^2^)	23.9 (23.2-24.5)	23.9 (23.3-24.6)	1.0
Parity (n primiparous (%))	84 (48.0%)	109 (56.5%)	0.1
Offspring Sex (n females (%))	96 (54.9%)	81 (42.28%)	0.02
Twin pregnancies	2	0	0.2

The comparator group are women who reported no nausea or vomiting during pregnancy. Data are geometric means (95% confidence intervals) or numbers of participants.

### Maternal GDF15 concentrations

In the Cambridge Baby Growth Study the median GDF15 concentration was 11,004 pg/mL (range 2,378–34,621) in serum samples collected at mean gestational age 15.1 weeks (range 12.0–18.0). Maternal GDF15 concentrations were not associated with gestational age at sampling (linear model with log-GDF15: P=0.4, standardized β=-0.03). In the NIPTeR Study the median GDF15 concentration was 11,014 pg/mL (range 4,106–37,194, n=233) in plasma samples collected at mean gestational age 12.1 weeks (range 10.0–16.1).

### Maternal GDF15 concentrations and associations with nausea and vomiting in pregnancy

Maternal GDF15 concentrations around week 15 were higher in women who reported vomiting in the second trimester of pregnancy compared to those who reported no nausea or vomiting during pregnancy (P=0.02; Table 2). This association was unaltered by adjustment for gestation at serum sampling or by maternal BMI. There was no significant elevation of GDF15 concentrations in women reporting nausea alone in the second trimester or in women reporting nausea or vomiting in the first or third trimesters ( Supplementary Table 2).

**Table 2.** Maternal GDF15 concentrations by self-reported vomiting in the second trimester or antiemetic use during pregnancy. This table shows comparisons of circulating maternal GDF15 concentrations around week 15 of pregnancy in those women who reported nausea alone or vomiting in the second trimester of pregnancy, those women who reported taking antiemetics during pregnancy and those women who reported no nausea or vomiting in pregnancy in the Cambridge Baby Growth Study. These concentrations were raised in women who reported vomiting whether unadjusted or adjusted for gestational age without or without BMI. Adjusted levels were also higher in women who took antiemetics during pregnancy. No apparent differences were observed in women who reported nausea alone.

Group	n	Serum GDF15 Concentration (pg/mL)	Unadjusted	Adjusted for gestational age	Additionally adjusted for maternal BMI
No nausea or vomiting	193	10,593 (10,066-11,147)	Ref	Ref	Ref
Nausea without vomiting (second trimester)	325	10,772 (10,328-11,235)	P=0.6	P=0.6	P=0.5
Vomiting (second trimester)	175	11,581 (10,977-12,219)	P=0.02	P=0.02	P=0.02
Antiemetic use (any trimester)	11	13,157 (10,558-16,394)	P=0.06	P=0.04	P=0.04

Data are geometric means (95% confidence intervals).

Eleven women (1.4%) took antiemetics, ten of whom reported vomiting and one of whom reported nausea without vomiting. Their serum GDF15 concentrations were also raised compared to women who reported no nausea or vomiting during pregnancy (P=0.04, adjusted for gestation; Table 2).

### Maternal GDF15 concentrations and associations with prenatal screening markers

**Figure 1.**
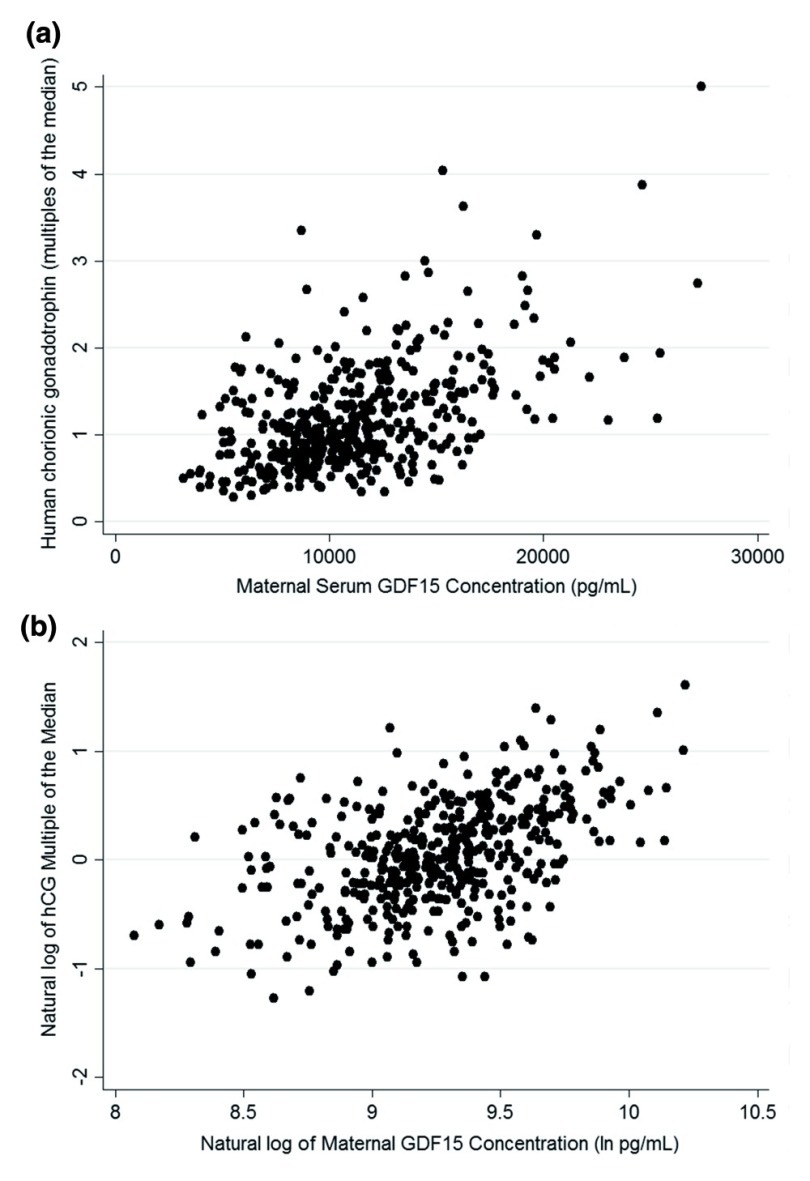
The relationship between week 15 maternal serum GDF15 concentrations and week 14 hCG MOMs. ( **a**) A scatter plot of untransformed GDF15 concentration and hCG MOM data from around weeks 14–15 of pregnancy in the Cambridge Baby Growth Study, ( **b**) a scatter plot of logarithmically-transformed data from the same cohort.

A subset of participants (441) had undergone a 14 week triple test at ~14 weeks gestation measuring AFP, estriol and hCG. Maternal serum GDF15 concentrations were not associated with AFP (standardized β=0.059, P=0.2, n=441). There was a weak positive association with unconjugated estriol (standardized β=0.110, P=0.02, n=440). In contrast there was a strong positive association with hCG ( Figure 1; standardized β=0.436, P=7.2×10 ^-22^, n=441). In contrast to the GDF15 data however, hCG levels were not significantly higher in women reporting vomiting in the second trimester of pregnancy: no nausea or vomiting 1.06 (0.96-1.17) (n=119) v. 1.17 (1.04-1.31) (n=91) (P=0.2).

### Maternal GDF15 concentrations and associations with pre-pregnancy BMI

**Figure 2.**
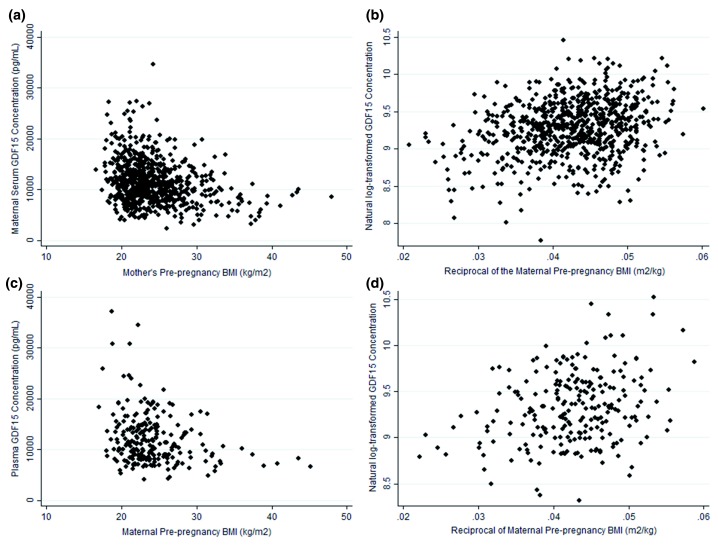
The relationship between maternal serum GDF15 concentrations around week 15 of pregnancy and pre-pregnancy BMIs. ( **a**) A scatter plot of untransformed GDF15 concentrations from around week 15 of pregnancy and pre-pregnancy BMI data from the Cambridge Baby Growth Study, ( **b**) a scatter plot of transformed data from the same cohort, ( **c**) a scatter plot of untransformed GDF15 and BMI data from the NIPTeR Study and ( **d**) a scatter plot of transformed data from the same study.

In the Cambridge Baby Growth Study the distribution of the relationship of week 15 GDF15 concentrations and maternal pre-pregnancy BMI was asymptotic ( Figure 2a), with higher GDF15 levels being seen exclusively in leaner mothers. The data were analyzed using log transformation of GDF15 levels ( Figure 2b) and a highly significant relationship with the reciprocal of pre-pregnancy BMI was apparent (standardized β=0.266, p=4.1×10 ^-13^, n=721).This relationship was replicated in the NIPTeR Study ( Figure 2c & d; standardized β=0.280, p=1.5×10 ^-5^, n=231).

## Discussion

In this large prospective pregnancy cohort study, maternal circulating GDF15 concentrations around week 15 of pregnancy were higher in women who reported vomiting in the second trimester and were even higher in women who reported taking antiemetics during pregnancy, compared to those of women who reported no nausea or vomiting during pregnancy. The results from the women who took antiemetics during pregnancy probably reflect the severity of their symptoms rather than their treatment. We also found that week 15 GDF15 concentrations were related to maternal pre-pregnancy BMIs, with the highest circulating GDF15 concentrations found in mothers with the lowest BMIs.

To our knowledge, this is the first report relating GDF15 concentrations to vomiting during pregnancy. Circulating GDF15 concentrations rise rapidly in maternal blood during early pregnancy and several studies have reportedly substantially lower concentrations at around 6–13 weeks gestation in those pregnancies that subsequently miscarried ^29, 30^. Possible explanations for this highly reproducible phenomenon have included the suggestion that maternal circulating GDF15 is a biomarker of successful placentation. Alternatively it has been suggested that GDF15 may promote fetal viability through an immunomodulatory action ^31^. However the recent discovery of the highly specific expression of the receptor for GDF15 in the hindbrain makes this less likely ^32^. Despite that uncertainty, our findings provide a possible mechanistic explanation for the widely observed associations between NVP and lower rates of miscarriage ^33^.

There are at least three possible interpretations of the inverse association between serum GDF15 concentrations and maternal pre-pregnancy BMI which we found in two independent studies. It is possible that those women who develop high levels of GDF15 in pregnancy have an intrinsic tendency to be GDF15 overproducers. Even in the non-pregnant state, given the known effects of this hormone on appetite ^34^ this could be directly related to their low weight. Such a hypothesis would need to take into account the fact that a substantial amount of the GDF15 found in maternal blood is likely to be secreted by the trophoblast, which is fetally-encoded and only shares ~50% genetic identity with the mother. An alternative interpretation is that women with a low pre-pregnancy BMI are particularly vulnerable to the stressful effects of pregnancy. Consistent with this GDF15 appears to be overproduced by a variety of tissues in response to different stress states, including undernutrition ^35^. Finally our findings could be consistent with the idea that early placentation is less successful in women with higher BMIs. This notion is supported by the fact that there is a graded increase in pregnancy loss with increasing maternal BMI ^36^, as well as an increase in maternal placental syndromes, including preeclampsia and gestational hypertension as maternal BMI increases ^37^. However this also has to be contrasted with the increased birth weights of babies born to women with high BMIs.

In the current study we found a remarkably strong association between GDF15 concentrations around week 15 of pregnancy and week 14 hCG levels. Both are major endocrine products of the placenta and it is likely that they at least in part reflect the functional mass of placenta. Fewer women has hCG measured in our study which may explain why we did not demonstrate a relationship of hCG levels and symptoms. Although it the most widely implicated hormone thought to stimulate NVP, hCG’s link to NVP is inconsistent ^13^ and largely relates to associations between the timing of changes in its concentrations and NVP symptoms rather than to a known pathogenesis ^24^. Given that there is a potential mechanism linking GDF15 concentrations in pregnancy with vomiting ^38^, the results from the present study raise the possibility that GDF15 is actually the causal factor (or at least one of them) and that reported associations between hCG and NVP ^24^ actually reflect GDF15 concentrations and bioactivity. These findings require confirmation in other studies.

Although we have uniquely shown associations between circulating GDF15 concentrations and vomiting in pregnancy, pre-pregnancy BMI and circulating hCG levels, this study has a number of limitations. Ideally, we would have measured maternal GDF15 concentrations in samples collected at gestational age 9 weeks, which is the peak for NVP symptoms ^39^. However, many women have not yet presented to maternal health services at that stage, and indeed for many women NVP represents the first indication of pregnancy. A further limitation is that maternal BMI was only available pre-pregnancy although weight is unlikely to have changed much during the initial 15 weeks of pregnancy. Finally and unsurprisingly, reflecting recruitment of the cohort at routine antenatal clinics, cases of HG were under-represented. Future case-control study designs are therefore needed to test whether our findings can be extrapolated to HG.

On the basis of a substantial body of recently emerging data we have previously proposed that the role of GDF15 in the adult organism is to provide a signal to the brain that the organism is engaging in damaging behavior ^38^. Its hindbrain-localized receptor activates a signal which is likely to be aversive and promote the future avoidance of this particular behavior. We propose that the placenta has evolved to use the GDF15 system to promote a state in which the mother is sensitized to other adverse stimuli, particularly those that might come from food, in order to protect the fetus from exposure to maternal ingestion of potential teratogens during the vulnerable stages of organ development. In the context of the recently revealed biology of GDF15 these data suggest that antagonism of GDF15 may have some potential for therapeutic benefit in NVP.

## Data availability

Open Science Framework: Data for associations of vomiting and antiemetic use in pregnancy with levels of circulating GDF15 early in the second trimester: A nested case-control study. https://doi.org/10.17605/OSF.IO/5JT3K ^40^.

Data are available under the terms of the Creative Commons Zero "No rights reserved" data waiver (CC0 1.0 Public domain dedication).

Data are present from both the Cambridge Baby Growth and the NIPTeR Studies, merged from the individual central record databases for each study. Anonymized data for the Cambridge Baby Growth study are available to other investigators through collaborative agreements, and the co-investigators welcome formal or informal proposals and will consider these at their bimonthly meetings. Please contact Dr Carlo Acerini [ cla22@cam.ac.uk]. Concerning NIPTeR study data, please contact Dr. Gijs Afink [ g.b.afink@amc.uva.nl].

## Supplementary material

**Supplementary Tables**: Clinical characteristics of women in the Cambridge Baby Growth Study who filled in and returned their questionnaires in comparison to those that did not, and week 15 GDF15 concentrations in women who reported nausea or vomiting in the first or third trimesters of pregnancy are appended.
