# Supplementary material for: Associations of vomiting and antiemetic use in pregnancy with levels of circulating GDF15 early in the second trimester: A nested case-control study
Source: Wellcome Open Res. 2018 Sep 21;3:123. [Version 1] doi: 10.12688/wellcomeopenres.14818.1 (PMC6171563; doi:10.12688/wellcomeopenres.14818.1)
Supplement: Supplementary file 1 [file wellcomeopenres-3-16147-s0000.tgz › 263654a9-2159-4efa-81c5-7f0f8a168b50_Supplementary_Tables.docx]

**Supplementary Tables for:**

**Associations of Vomiting and Anti-Emetic Use in Pregnancy with**

**Levels of Circulating GDF15 early in the Second Trimester**

Clive J. Petry^1^ , Ken K. Ong^1,2^, Keith A. Burling^3^, Peter Barker^3^, Sandra F. Goodburn^4^, John R.B. Perry^2^, Carlo L. Acerini^1^, Ieuan A. Hughes^1^, Rebecca C. Painter^5^, Gijs B. Afink^6^, David B. Dunger^1,7^, Stephen O’Rahilly^4,7^

^1^Department of Paediatrics, University of Cambridge, Addenbrooke’s Hospital, Cambridge CB2 0QQ, U.K.

^2^Medical Research Council Epidemiology Unit, Wellcome Trust-MRC Institute of Metabolic Science, University of Cambridge, Addenbrooke’s Hospital, Cambridge CB2 0QQ, U.K.

^3^NIHR Biomedical Research Centre Core Biochemistry Assay Lab, Department of Clinical Biochemistry, Addenbrooke’s Hospital, Cambridge CB2 0QQ, U.K.

^4^Department of Clinical Biochemistry, Addenbrooke’s Hospital, Cambridge CB2 0QQ, U.K.

^5^Department of Gynaecology and Obstetrics, Academic Medical Center, Amsterdam, The Netherlands

^6^Reproductive Biology Laboratory, Academic Medical Center, Amsterdam, The Netherlands

^7^Metabolic Research Laboratories and MRC Metabolic Diseases Unit, Wellcome Trust-MRC Institute of Metabolic Science, University of Cambridge, Addenbrooke’s Hospital, Cambridge CB2 0QQ, U.K.

**Supplementary Table 1** Clinical characteristics of those women that returned and those that did not return prenatal questionnaires.

| **Clinical Characteristic** | **Returned Questionnaire** | **Non-Returned Questionnaire** | **p‑value** |
| --- | --- | --- | --- |
| Pre-pregnancy BMI (kg/m^2^) | 24.1  (23.8, 24.4)  (n=1098) | 24.1  (23.2, 25.1)  (n=86) | 0.9 |
| Parity (n primiparous (%)) | 495 (45.1%) | 37 (43.0%) | 0.6 |
| Offspring Sex (n females (%)) | 520 (48.8%) | 42 (47.4%) | 0.8 |
| Adjusted offspring birth weight (kg) | 3.482  (3.456, 3.508)  (n=1096) | 3.403  (3.310, 3.497)  (n=86) | 0.1 |

Data are mean (95% confidence interval). Birth weights were adjusted for maternal pre-pregnancy BMI, gestational age at birth, offspring sex and parity.

**Supplementary Table 2** Maternal GDF15 concentrations around week 15 of pregnancy by self-reported vomiting in the first or third trimester of pregnancy.

| **Group** | **n** | **Serum GDF15 Concentration (pg/mL)** | **Unadjusted** | **Adjusted for gestational age** | **Additionally adjusted for maternal BMI** |
| --- | --- | --- | --- | --- | --- |
| No nausea or vomiting throughout pregnancy | 193 | 10,593 (10,066-11,147) | Ref | Ref | Ref |
| Nausea without vomiting (first trimester) | 616 | 10,941 (10,609-11,284) | P=0.09 | P=0.08 | P=0.2 |
| Vomiting (first trimester) | 291 | 11,277 (10,774-11,805) | P=0.08 | P=0.08 | P=0.1 |
| Nausea without vomiting (third trimester) | 50 | 10,284 (9,224-11,464) | P=0.4 | P=0.4 | P=0.4 |
| Vomiting (third trimester) | 38 | 10,648 (9,333-12,148) | P=0.9 | P=0.9 | P=1.0 |

Data are geometric means (95% confidence intervals).
