# Supplementary material for: Associations of vomiting and antiemetic use in pregnancy with levels of circulating GDF15 early in the second trimester: A nested case-control study
Source: Wellcome Open Res. 2018 Sep 21;3:123. [Version 1] doi: 10.12688/wellcomeopenres.14818.1 (PMC6171563; doi:10.12688/wellcomeopenres.14818.1)
Supplement: Peer review history

# Peer review: Referee response for version 1

**DOI:** 10.21956/wellcomeopenres.16147.r33961

**Published:** 2018-10-19

**Authors:** Samuel N. Breit

**Affiliations:** 1 St Vincent's Centre for Applied Medical Research, St Vincent’s Hospital, Sydney, NSW, Australia

This paper utilises prospectively collected samples from two separate cohorts to evaluate the relationship of serum GDF15 levels to nausea, vomiting and anti-emetic medication use during early pregnancy.  Within the limitations clearly identified by the authors, this is a well conducted study and the results were largely consistent across both cohorts. The data identified a significant relationship between second trimester vomiting or anti-emetic drug use during pregnancy, with serum GDF15 levels. However, no similar relationship was identified, in pregnant women who had nausea alone.  Interestingly, GDF15 serum concentrations during the pregnancy were inversely correlated with pre-pregnancy maternal BMI’s across, both cohorts.  The mechanisms for this is currently unclear, but raises interesting questions about the biology of GDF15 in pregnancy. 

 The main limitations of this study, recognized by the authors, are those inherent in using samples from a study, which was not specifically designed to address the role of GDF15 in pregnancy.  The importance of this study is that it provides the first biologically plausible data to explain this very common and sometimes serious pregnancy complication, and the first clinical validation for the recently published genetic data, cited by the authors, linking a GDF15 polymorphism to the most severe form of pregnancy associated vomiting, hyperemesis gravidarum.  This paper should provide significant impetus for studies specifically directed to the role of this cytokine in pregnancy including its effects on foetal growth and post-partum development.

I have read this submission. I believe that I have an appropriate level of expertise to confirm that it is of an acceptable scientific standard.

# Peer review: Referee response for version 1

**DOI:** 10.21956/wellcomeopenres.16147.r33960

**Published:** 2018-10-03

**Authors:** Jone Trovik

**Affiliations:** 1 Bergen Gynaecologic Cancer Research Group, Department of Clinical Medicine, University of Bergen, Bergen, Norway

The authors here present an interesting and well conducted study using prospectively collected blood samples from two different cohorts of women enrolled during early 2nd trimester. Self-reported pre-pregnant weight, height and nausea and vomiting or use of antiemetics were used to calculate BMI and categorize severity of NVP (nausea and vomiting of pregnancy). Blood samples were analysed for HCG (the Dutch NIPTeR cohort only) and GDF-15 (both cohorts). Levels of GDF-15 were correlated to severity of NVP, pre-pregnant BMI and HCG levels.

 GDF-15 levels were found to be higher for women reporting vomiting compared to women with no NVP, higher for women with low pre-pregnant BMI and significantly correlated to HCG-levels.

 The rationale for conducting the study, methodological conduct and discussion is well presented.

 The main weakness is that the report of NVP system is retrospective, and as I understand for the Cambridge study; the questionnaire was returned after delivery. Although the women were asked to fill inn “real-time” (from enrolment at 15-weeks) the authors cannot account for the proportion of women actually reporting real-time.

 The report of NVP during first trimester was retrospective for all women.

 The authors have not used a validated NVP-questionnaire. The PUQE-questionnaire has been validated also for use reporting retrospectively the extent of NVP during the whole of first trimester. In addition this would have yielded a scale, with possibility of a linear comparison of NVP-grade versus GDF-15 level.

 Another weakness (also acknowledged by the authors) is that the peak of NVP-symptoms usually occurs during first trimester, while women here are investigated during their second trimester. Also as pointed out: Hyperemesis gravidarum occurs in approximately 1% of pregnancies, thus if aiming to investigate women with this disease a general pregnancy cohort is not feasible.

 It would have added strength to the study if they had reported weight at inclusion/weigh-change from pre-pregnancy. As they introduce GDF-15 as related to appetite/food suppression a correlation to weight-change could have added to that discussion.

I have read this submission. I believe that I have an appropriate level of expertise to confirm that it is of an acceptable scientific standard.

# Response

**Published:** 2018-10-03

**Authors:** Clive Petry

**Affiliations:** Dr, UK

We thank the reviewer for her helpful suggestions in reviewing our manuscript. In terms of our questionnaire she is correct that the study participants were encouraged to fill them in as their pregnancies progressed but they were not collected by research staff until after the birth of their babies. Clearly these data were compiled as part of a prospective, observational cohort study that was not set up to specifically test the hypothesis stated in this manuscript. We acknowledge that this is a limitation of the current study.

# Peer review: Referee response for version 1

**DOI:** 10.21956/wellcomeopenres.16147.r33959

**Published:** 2018-09-25

**Authors:** Roger Gadsby

**Affiliations:** 1 Warwick Medical School, University of Warwick, Coventry, UK

This is a prospective pregnancy cohort study showing that maternal circulating GDF15 concentrations at around week 15 in pregnancy were higher in women who reported vomiting in the second trimester compared to women who reported no nausea.

 It is a well conducted study using data from women  and blood samples from women who took part in well known, clearly defined prospective studies. The number of women reporting no NVP symptoms is in line with published data.

 This paper is the first to relate GDF15 concentrations to vomiting during pregnancy and as such is of considerable interest. The aetiology of nausea and vomiting in pregnancy is as yet unknown and so associations between symptoms and this newly emerging compound GDF15 are fascinating.

 The weaknesses of the study are clearly stated in the discussion. Some are related to the timing of the samples and information about NVP symptoms at 15 weeks when the peak week for symptoms is week 9 from LMP. This means that much information about the NVP symptoms would be retrospective through the women having to remember what she felt like 4-6 weeks previously. Recall may be incomplete and biased towards forgetting symptom severity once the symptoms improve.

 In most studies of NVP symptoms there is a clear relationship between nausea and vomiting in that vomiting only occurs in women with severe nausea. The fact that GDF15 is not raised in women reporting nausea without vomiting compared to those with no vomiting is not what I would have expected.

I have read this submission. I believe that I have an appropriate level of expertise to confirm that it is of an acceptable scientific standard.
